# Role of transcription factors in porcine reproductive and respiratory syndrome virus infection: A review

**DOI:** 10.3389/fmicb.2022.924004

**Published:** 2022-07-19

**Authors:** Xiangbin You, Ying Lei, Ping Zhang, Dequan Xu, Zulfiqar Ahmed, Youbing Yang

**Affiliations:** ^1^College of Animal Science and Technology, Henan University of Science and Technology, Luoyang, China; ^2^Luoyang Key Laboratory of Animal Genetics and Breeding, Luoyang, China; ^3^College of Animal Science and Technology, Huazhong Agricultural University, Wuhan, China; ^4^Faculty of Veterinary and Animal Sciences, University of Poonch Rawalakot, Rawalakot, Pakistan

**Keywords:** transcription factors, PRRSV, miRNA, immune evasion, immune cell, phosphorylation, nuclear localization

## Abstract

Porcine reproductive and respiratory syndrome (PRRS) is an infectious disease caused by the PRRS virus that leads to reproductive disorders and severe dyspnoea in pigs, which has serious economic impacts. One of the reasons PRRSV cannot be effectively controlled is that it has developed countermeasures against the host immune response, allowing it to survive and replicate for long periods. Transcription Factors acts as a bridge in the interactions between the host and PRRSV. PRRSV can create an environment conducive to PRRSV replication through transcription factors acting on miRNAs, inflammatory factors, and immune cells. Conversely, some transcription factors also inhibit PRRSV proliferation in the host. In this review, we systematically described how PRRSV uses host transcription factors such as SP1, CEBPB, STATs, and AP-1 to escape the host immune system. Determining the role of transcription factors in immune evasion and understanding the pathogenesis of PRRSV will help to develop new treatments for PRRSV.

## Introduction

Porcine reproductive and respiratory syndrome (PRRS) is a disease caused by PRRSV that leads to typical reproductive disorders and respiratory diseases in pigs (Teuffert et al., 1998; Montaner-Tarbes et al., 2019). PRRS affects pigs at each stage, causing slow growth in finishing pigs, premature birth and miscarriage in sows and poor semen quality in boars (Schulze et al., 2013; Olanratmanee et al., 2015; Helm et al., 2019). PRRS has greatly affected the U.S. pig industry, which has an annual worth of $664 million, since it was first identified (Holtkamp et al., 2013; Pileri and Mateu, 2016). It is considered one of the major diseases threatening the pig industry globally (Butler et al., 2014; Dhakal and Renukaradhya, 2019).

**Figure 1:**
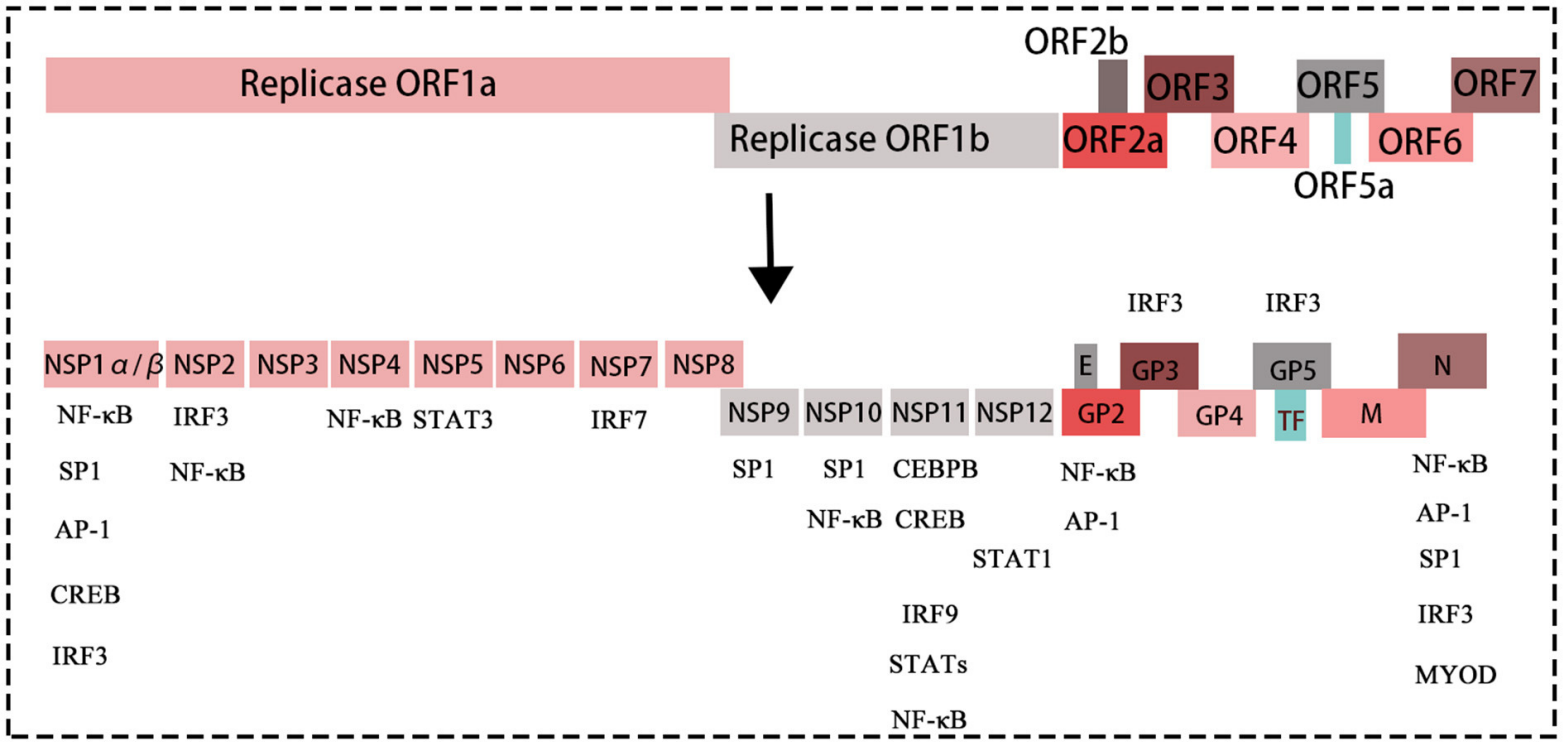
The structure of the PRRSV genome, with different colored boxes representing different genes of the virus. The proteins translated by each gene of PRRSV. The genes without boxes below are transcription factors, which can bind/interact with structural proteins and non-structural proteins of PRRSV, thus affecting replication of PRRSV.

PRRSV is a single-stranded positive-sense enveloped RNA virus belonging to the genus *Arteritis* (Dokland, 2010). The virus particles are spherical with a diameter of 55–60 nm. The entire length of the genome of PRRSV is ~15.4 kb and contains a 5' cap and 3' poly tail structure. The PRRSV genome contains at least 11 open reading frames (ORFs): ORF1a, ORF1b, ORF2a, ORF2b, ORF3, ORF4, ORF5, ORF6, ORF7, ORF5a, and ORF2 (TF) (Johnson et al., 2011) (Figure 1). Among these ORFs, ORF1a and ORF1b account for ~75% of the viral genome, encoding the proteins with apparent replication and polymerase activities. ORF1a and ORF1b encode two large non-structural polyproteins (pp1a and pp1ab) that play essential roles in the replication process and are subsequently hydrolysed into 14 non-structural proteins (Li et al., 2014). Recently, the role of the non-structural proteins (nsps: nsp1, nsp2, nsp4, and nsp11) in the regulation of the host immune response after PRRSV was explored (Fang and Snijder, 2010; Dong et al., 2018; Jing et al., 2019). ORF2–7 are expressed from six subgenomic mRNAs, which encode eight structural proteins, which include nucleocapsid protein (N) and minor (GP2a, GP3, GP4, E, and ORF5a) and major (GP5 and M) envelope proteins. The N protein has the ability to inhibit interferon (Yoo et al., 2010) whereas GP2, GP3, and GP4 form multiprotein complexes during PRRSV infection by receptor binding (Das et al., 2010).

PRRSV only infects pigs, is characterized by a specific tropism to differentiated macrophages and shows a minimal ability to infect mononuclear cell lines (Duan et al., 1997a; Lunney et al., 2016). In addition, dendritic cells have also been reported to support PRRSV replication (Duan et al., 1997a; Loving et al., 2007). PRRS infection is divided into the three stages of acute infection, persistent infection, and regression. In the acute infection stage, PRRSV replicates mainly in macrophages and dendritic cells of the lung and upper respiratory tract, resulting in viremia by 6–12 h postinfection. The stage of persistent infection is divided into two phases: the first phase is the peak of replication after 2 weeks of infection, and the second stage is a lower level of replication during the 5th to 7th weeks of infection (Duan et al., 1997b; Labarque et al., 2000). At this stage, virus replication is mainly confined to the lymphatic organs (tonsils and lymph nodes) (Rowland et al., 2003). PAMS, the first line of defense, are destroyed by apoptosis within 2 weeks of PRRS infection, which increases the mortality of infected pigs (Thacker, 2001; Ouyang et al., 2019). PRRSV-infected pigs mainly show mild to severe interstitial pneumonia, which later manifests as purulent bronchopneumonia. Extensive bleeding and edema in the lungs leads to respiratory failure in infected pigs (Morgan et al., 2016). The final disappearance of the virus is the regression stage of infection and it is not clear how long the PRRS infection will take to subside, although replication of the virus and maintenance of PRRS can extend up to 250 days following infection (Wills et al., 2003).

PRRS has not been effectively controlled because of two major characteristics of PRRSV (Du et al., 2017). First, PRRSV is a virus prone to mutation and has many strains, which has resulted in vaccines that protect against homologous strains in vaccinated animals but have limited protection against heterogenic strains (Charerntantanakul, 2012; Renukaradhya et al., 2015). Second, PRRSV has developed a countermeasures against the host's immune system that helps it survive and replicate for a long time before the infection diminishes. In the process of evolution, the continuous interaction between the virus and host forms and determines their survival strategy (Murtaugh et al., 2002; Ma et al., 2021). To replicate and spread in the host, the virus has evolved a variety of immune escape mechanisms, resulting in the subversion of a variety of cellular immune signaling pathways (Du et al., 2017; Wang et al., 2021).

TFs are proteins that control gene expression by binding to specific DNA sequences and thereby control gene transcription *via* the up- and downregulation of RNA polymerase or other regulatory proteins (Jolma et al., 2013). Transcription factors play an important role in the interaction between the host and PRRSV, whether by helping the virus escape host immunity or by inhibiting PRRSV replication. In the present review, we systematically described how PRRSV uses host transcription factors to escape the host immune system. Determining the role of transcription factors in immune evasion and understanding the pathogenesis of PRRSV will help to develop new treatments for PRRSV.

## The function of transcription factors

TFs account for ~8% of all human genes and are the center of a wide regulatory network that regulates gene expression and triggers different biological responses (Lambert et al., 2018). TFs usually regulate gene expression by combining enhancer elements and recruiting coactivators and RNA polymerase II (Lelli et al., 2012; Spitz and Furlong, 2012). It is worth mentioning that TFs can enhance the expression of a gene from very remote positions even from several thousand base pairs by up- or downstream of transcription initiation sites. The core promoter elements also consist of sites where transcription initiation occurs, which certain TFs bind to. The regulation of gene expression by TFs is very complex, and the transcriptional expression of many genes requires single to multiple TFs. The mammalian TF NF-κB consists of P50, P52, REL, Rel-A, and Rel-B, and these proteins dimerize to form functional NF-κB, which is due to the specific expression pattern of TFs expressed by specific genes of specific cell types at specific times (Stampfel et al., 2015). In addition, the role of TFs in gene transcription is uncertain; they can act as protein activators or suppressors due to the presence (or absence) of other TFs.

TFs are present in virtually all cell types and are associated with various cellular processes, such as cell proliferation, cell differentiation, and host immune responses. Considering the complexity and dynamic nature of the TF network, these processes are easily disturbed by dysfunctional TFs, leading to the occurrence and development of diseases. The deregulation of TFs is characteristic of most human cancers, and a classic example is the tumor suppressor gene p53, which is deregulated in more than half of human cancers (Khoo et al., 2014). TFs are at the core of multiple signaling pathways in eukaryotic cells; therefore, these proteins must be targeted more specifically than traditional targets. TF genes of the same family or from different eukaryotic organisms show similar structural and functional features, thus indicating the evolution of TFs from a common ancestor. Some conserved features or sequences of TFs are found in eukaryotes, including the TF dimerization network and TF DNA binding motif preference.

## The role of transcription factors in PRRSV infection

### Transcription factors regulate PRRSV replication through miRNAs

In mammals, miRNAs control ~50% of the activity of protein-coding genes and participate in the regulation of cellular activities. MiRNAs are widely expressed in a variety of innate immune cells (PAMs, NK cells, and DCs), which are the first line of defense during the occurrence of infections. MiRNAs also play a crucial role in mediating host acquired immunity; however, their abnormal expression results in the manifestation of diseases (Krol et al., 2010). TFs and miRNAs are the two main types of gene regulators, which jointly regulate gene expression at the transcriptional and posttranscriptional levels and control each other's expression. The regulatory network formed between TFs and miRNAs is considered an effective method for analyzing and studying the complexity of biological regulation (Barabasi and Oltvai, 2004). During viral infection, many viral elements act on TFs which in turn negatively regulate the immune response and promote viral replication.

#### SP1

Specific protein 1 (SP1) is a well-known member of the transcription factor family that is involved in a large number of important biological processes and can activate the transcription of many cellular genes (Vizcaino et al., 2015). Studies have shown that PRRSV nsp9 and N promote the expression of miR-373 through SP1, and miR-373 directly regulates the expression of nuclear factor IA (NFIA) and nuclear factor IA (NFIB), which inhibits the expression of IFN-β and ultimately PRRSV replication. The upregulation of miR-373 expression can promote the replication of PRRSV (Chen J. et al., 2017). In conclusion, PRRSV regulates miRNA by influencing the expression of transcription factors, which in turn affects host-related gene expression and creates an optimal environment for virus replication.

#### IRF7

Interferon regulatory factors (IRFs) are a family of transcription factors that includes 9 members. It regulates many aspects of innate and adaptive immune responses, including driving antiviral responses, responding to pathogens, driving proinflammatory responses and regulating immune cell differentiation (Jefferies, 2019). Studies have found that PRRSV-2 significantly increases the expression of miR-541-3p and inhibits the expression of transcription factor interferon regulatory factor 7 (IRF7) after infecting Marc-145 cells. Additional, miR-541-3p negatively regulates the transcription of type I interferon by targeting IRF7, resulting in evasion of the host immune response by PRRSV and promotion of virus replication (Shi et al., 2022). This indicates that PRRSV-2 inhibits the host innate immune response by hijacking host miR-541-3p, and the role of transcription factors in this process cannot be denied.

#### IRF1

IRF1 is involved in various physiological and pathological aspects, including viral infection, proinflammatory injury and the development of the immune system. Studies have shown that overexpression of miR-296-3p can promote the replication of PRRSV by inhibiting the expression of IRF1 and TNF-α. TNF is a pleiotropic cytokine that is closely related to inflammation and innate and adaptive immune responses to pathogens. Notably, IRF1 regulates the expression of TNF-α by activating the TNF promoter *via* IRF1 response elements (Zhang et al., 2021). TNF may be involved in IFN-β induction. TNFR1 binding mediates IRF1, and IFN-β expression is increased by prolonging the expression of proinflammatory chemokines through STAT1 (Feng et al., 2021). HP-PRRSV infection activates the IRF1/TNF-α signaling axis in PAMs by downregulating host miR-296-3p. In addition, there may be host factors involved in regulating the downregulated expression of miR-296-3p, which inhibits replication of the virus *in vivo*.

#### IRF8

miR-10a has been found to be significantly overexpressed in PRRSV-infected PAM cells and inhibit viral replication by inhibiting host molecule signal-recognition particle 14 (SRP14) protein (Zhao et al., 2017). In another study, PRRSV infection reduced the expression level of IRF8 in PAMs, leading to upregulation of miR-10a, which played an anti-PRRSV role (Zheng et al., 2022). It is strange that the transcription factor IRF8 is necessary for the development and maturation of myeloid cells (dendritic cells, monocytes, macrophages) and the expression of their internal antimicrobial function (Salem et al., 2020). IRF8 plays an important role in preventing infection through the activities of several immune cell types. However, Zheng et al. (2022) found that the transcription factor IRF8 promoted PRRSV replication by inhibiting miR-10a.

#### STAT1

Our previous study showed that miR-210 directly targets STAT1 to inhibit the expression of the lung injure-related factors MCP-1, VCAM-1, and ICAM-1, while STAT1 also acts on the TNF-α promoter region to regulate its expression (You et al., 2020). Similar to our findings, a large number of studies have shown that the transcription factor STAT1 plays an important role in regulating the molecular mechanism of lung injury.

### Transcription factors exacerbate inflammation and injury, which responsibility for the additional morbidity and mortality of the infected pigs

PRRSV infection causes severe lung inflammation and injury with subsequent pulmonary edema, hemorrhage, pneumonia, and peribronchitis (Han et al., 2017). Elevation of proinflammatory cytokines in PRRSV-infected pigs is a part of the pathogenesis of PRRSV. Previous studies also demonstrated that the expression of IFNα/β, TNFα, IL-1β, IL-6, and IL-8 in serum, BALF and tracheobronchial lymph node homogenates increased after infection with PRRSV (Guo et al., 2013). Studies have shown that the likely cause of death after infection with HP-PRRSV is severe inflammatory damage, rather than uncontrolled infection. Transcription factors exacerbate lung inflammation and injury during PRRSV infection, which may be responsible for the additional morbidity and mortality in the infected pigs (Han et al., 2014).

#### CREB/CEBPB

CCAAT/enhancer binding protein (CEBPB) family transcription factors are closely related to inflammation observed in various viral and traumatic diseases. IL-17 is a proinflammatory cytokine that is closely related to the strong inflammation caused by PRRSV. Research has shown that PRRSV nsp11 is involved in IL-17 production and PRRSV replication. CEBPB exacerbates lung inflammation by promoting IL-17 production during induced PRRSV infection. In porcine IL-17 promoters, deletion of CEBPB and the CREB binding motif inactivated PRRSV-induced IL-17 production, while deletion of CEBPB and CREB significantly reduced PRRSV-induced IL-17 production, suggesting that IL-17 expression is dependent on CEBPB and CREB (Wang et al., 2019). In addition, CEBPB also plays a critical role in macrophage production, activation and polarization, and it has been observed that CEBPB knockout mice lack alveolar macrophages (Cain et al., 2013).

#### NF-κB

NF-κB belongs to a family of inducible transcription factors involved in pathogen- or cytokine-induced immune and inflammatory responses. Many viruses encode proteins that activate or modulate NF-κB signaling pathways for their own advantage (Santoro et al., 2003). The high expression of IL-15 is responsible for the influx of NK cells and cytotoxic T lymphocytes in the lungs, which in turn causes severe respiratory distress in infected pigs (Fu et al., 2012). Deletion analysis confirmed that the NF-κB motif is essential for the activation of the porcine IL-15 promoter. NF-κB also regulates TNF-α expression by binding to the TNF-α promoter (Subramaniam et al., 2010). Studies have shown that TNF-α promotes inflammation at the site of infection by inducing the production of other proinflammatory cytokines in the vicinity of infection (Toews, 2001).

#### AP-1

AP-1 is a ubiquitous family of dimeric transcription complexes involved in a plethora of cellular and physiological functions. AP-1 has also been associated with a variety of serious diseases, including organ damage and various inflammatory pathologies (Bejjani et al., 2019). IL-6 plays an important role in the inflammatory response. Overexpression of IL-6 can stimulate and activate the acute response leading to inflammation and injury. The porcine IL-6 promoter contains an AP-1 binding site, and the absence of the AP-1 binding site can impair PRRSV activation of the IL-6 promoter, suggesting that the expression of IL-6 depends on the activation of AP-1 (Xu et al., 2021). In summary, IL-6 or AP-1 may be possible targets for alleviating inflammation and injury caused by PRRSV infection.

### Phosphorylation and nuclear localization of transcription factors affect PRRSV replication

During PRRSV infection, the activation of specific TFs causes the translocation of these factors to the nucleus and initiates the transcription of genes encoding IFNs and proinflammatory cytokines in the nucleus. It is well known that type I IFNs play important roles in antiviral responses in both virus-infected and uninfected cells (Chen et al., 2016). The expression of type I IFN is influenced by many TFs, such as IRF3, IRF7, STATs and NF-κB.

#### IRF3

Innate immunity is an essential way for host cells to resist viral infection through the production of interferons (IFNs). Phosphorylation of IRF3 is a crucial step in the induction of IFNs (Tailor et al., 2006). Previously, it was observed that PRRSV non-tructural proteins (nsps) inhibit the production of IFN by affecting the phosphorylation and nuclear localization of IRF3. For instance, Li et al. showed that nsp2 strongly inhibited IFN-β production and IRF-3 phosphorylation and nuclear translocation (Beura et al., 2010; Li et al., 2010). IRF3 is constitutively expressed in most cell types and exists in the cytoplasm in an inactive form. Upon stimulation, IRF3 is phosphorylated and undergoes conformational changes leading to the dimerization, which reveals the nuclear localization signal and allows it to translocate to the nucleus (Lin et al., 1998; Dragan et al., 2007).

#### STATs

There are seven members of the STAT family in mammals. Phosphorylated STATs form homodimers or heterodimer complexes and are then translocated into the nucleus by importing proteins and bind with response elements in DNA to activate or inhibit the transcription of a specific group of genes (Yang and Zhang, 2017). nsp1β inhibits IFN-induced ISG expression by blocking the nuclear translocation of STAT1 (Patel et al., 2010). Similar to the nsps, structural proteins also affect the production of IFN, such as N and GP5 proteins (Sagong and Lee, 2011; Zhixuan et al., 2015). PRRSV inhibits the induction of type I interferon, which delays the development of neutralizing antibodies and deregulates cytokine expression. Interferon is an important antiviral molecule whose expression is triggered by pattern recognition receptors that recognize viral components through a series of signaling molecules that viruses can target to escape innate immunity. In addition, nsp12 of VRL-2385, a strain with moderate virulence, increases the expression of proinflammatory cytokines such as IL-1β and IL-8 by inducing STAT1 phosphorylation of serine 727 (pSTAT1-S727) in Marc-145 and PAMs (Yu et al., 2013).

#### RBM39

RNA binding motif protein 39 (RBM39) is a nuclear protein that is involved in precursor mRNA splicing. Studies have shown that PRRSV can significantly promote the expression of RBM39 in PRRSV-infected 3D4/21 cells (Song et al., 2021). The transcription factor RBM39 can evade the host immune system in the following ways: first, RBM39 alters the phosphorylation of the transcription factor c-Jun, and then inhibits the AP-1 pathway to promote virus differentiation; second, PRRSV infection causes RBM39 and c-Jun to migrate from the nucleus to the cytoplasm. The transcription factor c-Jun is expressed at low levels in a variety of cells and at high levels in response to various stimuli such as growth factors, cytokines, foreign substances and viral infection. In addition, RBM39 can bind to PRRSV (nsp4, nsp5, nsp7, nsp10-12, M and N) and promote PRRSV differentiation.

### Transcription factors regulate PRRSV replication through host immune cells

After PRRSV infection, activation of the immune cells of the host's innate immune system is the key link to prevent virus invasion and replication (Lunney et al., 2016). Immune cells are divided into specific or non-specific immune cells, and antigen-presenting cells. Non-specific immune cells include macrophages, neutrophils, natural killer cells, and mast cells. Whereas, specific immune cells include T cells and B cells. The antigen-presenting cells include dendritic cells (DCs), macrophages and B cells, and DCs are the most potent antigen-presenting cells of the immune system.

#### SP1 and NF-κB

Previous studies have shown that CD83 inhibits DC-mediated T-cell stimulation and interferes with DC cytoskeleton maturation, which participates in immunosuppressive responses *in vivo* (Chen et al., 2011). Interestingly, PRRSV infection significantly enhances the activity of the CD83 promoter in porcine monocyte derived DCs through the TFs SP1 and NF-κB thus avoiding the host immune defenses by inducing persistent infection (Chen X. et al., 2017).

#### T-Bet, Foxp3, and EOMES

CD4, CD25, and Foxp3 are important markers for identifying T-regulated lymphocytes (Tregs). Numerous reports have demonstrated that CD4+CD25+Tregs can inhibit host antiviral immune responses. Induction of CD4+CD25+ Tregs during an early stage of infection has been suggested to be the mechanism for developing chronic or persistent viral infections (Rouse et al., 2006). Earlier, it was found that PRRSV increases the number of virus-specific CD4^+^CD25^+^Foxp3^+^ Tregs *in vivo* and *in vitro* (Wongyanin et al., 2010). Foxp3 is a TF expressed by Tregs and its expression promotes viral load (Ferrarini et al., 2015). Another study showed that after infection with the Lena PRRSV strain, the expression of T-bet, Foxp3, and EOMES increased significantly, and at the same time, the expression level of IFN-γ also increased significantly. Importantly, the three significantly overexpressed transcription factors are closely related to the polarization of immune cells (Th1 cells, T cells, CD4+ cytotoxic T lymphocytes, and effector CD8+ T cells) (Ruedas-Torres et al., 2021).

#### AP-1

PRRSV exploits various strategies to influence host immune responses and the establishment of chronic persistent infections. Previous studies have shown that PRRSV induces the production of SOCS1 through TF AP-1 thereby inhibiting the expression of IFN-β and IFN-stimulated genes and promoting the replication of PRRSV (Luo et al., 2020). Intracellular SOCS protein is involved in the regulation of innate immunity and adaptive immunity, which negatively regulate the JAK/STAT and TLR signaling cascades, dendritic cell activation, T-cell differentiation and Th-cell regulation (Inagaki-Ohara et al., 2013; Linossi et al., 2018; Yoshimura et al., 2018).

### Other strategies for evading the host immune system that are regulated by transcription factors

#### TFDP2

Studies have found that some viruses can affect the process of the host cell cycle to provide a cell environment conducive to virus proliferation. It was found that TFDP2, a transcription factor significantly overexpressed in PRRSV-infected 3D4/21 cells, can positively regulate the expression of cyclin A, reduce the proportion of S-phase cells and promote the proliferation of PRRSV (Zhu et al., 2021). The reduction in the number of PRRSV cells in S phase was shown to be beneficial to PRRSV proliferation, possibly because the S phase provided fewer ribonucleotides for PRRSV RNA synthesis than the G0/G1 phase. This study provides a new mechanism by which PRRSV uses host proteins to regulate the cell cycle to escape the host immune system.

#### CEBPB

From the host perspective, previous studies have shown that pigs of different genetic lines exhibit different virus clearance capabilities and PRRSV resistances (Lunney and Chen, 2010), and TFs seem to explain the underlying genetic mechanism of PRRSV susceptibility differences. Moreover, Niu et al. reported that overexpression of CXCL14 inhibits PRRSV replication; in turn, PRRSV infection inhibits CXCL14 expression by downregulating the expression of TF CEBPB. The binding ability of the TF CEBPB to the CXCL14 promoter region of TongCheng pigs was weaker than that observed in LW pigs; therefore, the expression of CXCL14 in TongCheng pigs allowed the maintenance of high levels of PRRSV infection following exposure, and TongCheng pigs showed a certain resistance to PRRSV (Niu et al., 2020). In addition, high fever, which is one of the characteristics of PRRS during pathogen infection, and PGE2 are involved in inducing host hyperthermia. Bi et al. (2014) reported that HP-PRRSV infection increases the production of PGE2 by upregulating COX-1, and that the TF CEBPB works by binding to the COX-1 promoter region.

#### STAT2

PRRSV infection has little effect on STAT2 transcription levels, but interestingly, it can reduce STAT2 protein levels in a dose-dependent manner. Further studies have shown that PRRSV nsp11 can interact with STAT2, with the N-terminal domain (NTD) of nsp11 being responsible for STAT2 degradation, and also with the STAT2 NTD and core-coil domain (Yang et al., 2019). STAT2 plays a key role in the activation of host innate immune signals involving IFN-interferon. PRRSV nsp11 antagonizes IFN signaling by mediating STAT2 degradation, which provides new insights into PRRSV's avoidance of host immune responses. PRRSV can also induce STAT3 degradation through nsp5 and antagonize JAK/STAT3 signaling. Thus, PRRSV can spread and replicate rapidly in the host (Yang et al., 2017).

#### NRF2

In addition, natural products inhibit the replication of PRRSV through TFs. In this context, Liu et al. reported that Xanthohumol extracted from *Humulus lupus* L. significantly inhibited early PRRSV infection and virus-induced oxidative stress by activating the NRF2-mediated pathway in PAMs and Marc-145 cells (Liu et al., 2019).

## Transcription factors databases

### JASPAR

A database of TF binding profiles (Castro-Mondragon et al., 2021), and JASPAR is a free and public database of TFs that collects information on the binding sites and binding methods of TFs and DNA. The database collects the data of six different species of vertebrates, plants, insects, nematodes, fungi and caudal chords which can be used to predict the binding regions of TFs and sequences. In addition, there are nine subdatabases, each of which contains information from different sources and categories of TFs.

### TransmiR

This TF-microRNA regulation database (Tong et al., 2019) collects information on TF–miRNAs and identifies the regulatory relationship between TFs and miRNAs. This database contains information that is validated or obtained by ChIP-seq and predicts TF target genes. In addition, all TF-miRNAs were annotated in detail including enrichment analysis, disease-specific TF-miRNAs and other resources.

### AnimalTFDB

This is a comprehensive resource for annotation and prediction of animal TFs (Hu et al., 2019). The AnimalTFDB is a comprehensive database with 125,135 TF genes and 80,060 transcription cofactor genes at the genome-wide level of 97 species that have been identified, classified and annotated. TF was further divided into 73 families according to the DNA binding domain of TFs, and 83 families and 6 categories according to the function of TF cofactors. This database also provides a variety of search and browsing methods, two online prediction tools that predict TF and predict TFBS, blast tools and data download functions.

### hTFtarget

This is a comprehensive database for regulations of human TFs and their targets (Zhang et al., 2020). This database integrates 699 highly reliable DNA binding sequences of TFs, including 2,737 TFBS motifs of 699 TFs. It is the most comprehensive database of human TF targets at present. In addition to integrating large-scale human TF target gene data, an open-source human TF target gene database has been constructed. The cell line specific regulation of TFs and cooperative regulation among TFs have been analyzed, which provides a similar one-stop solution for research on TF target regulation. hTFtarget can be applied in two different strategies to detect TF target reliability, including chip SEQ data analysis and TF binding site scanning.

## Conclusions

**Figure 2:**
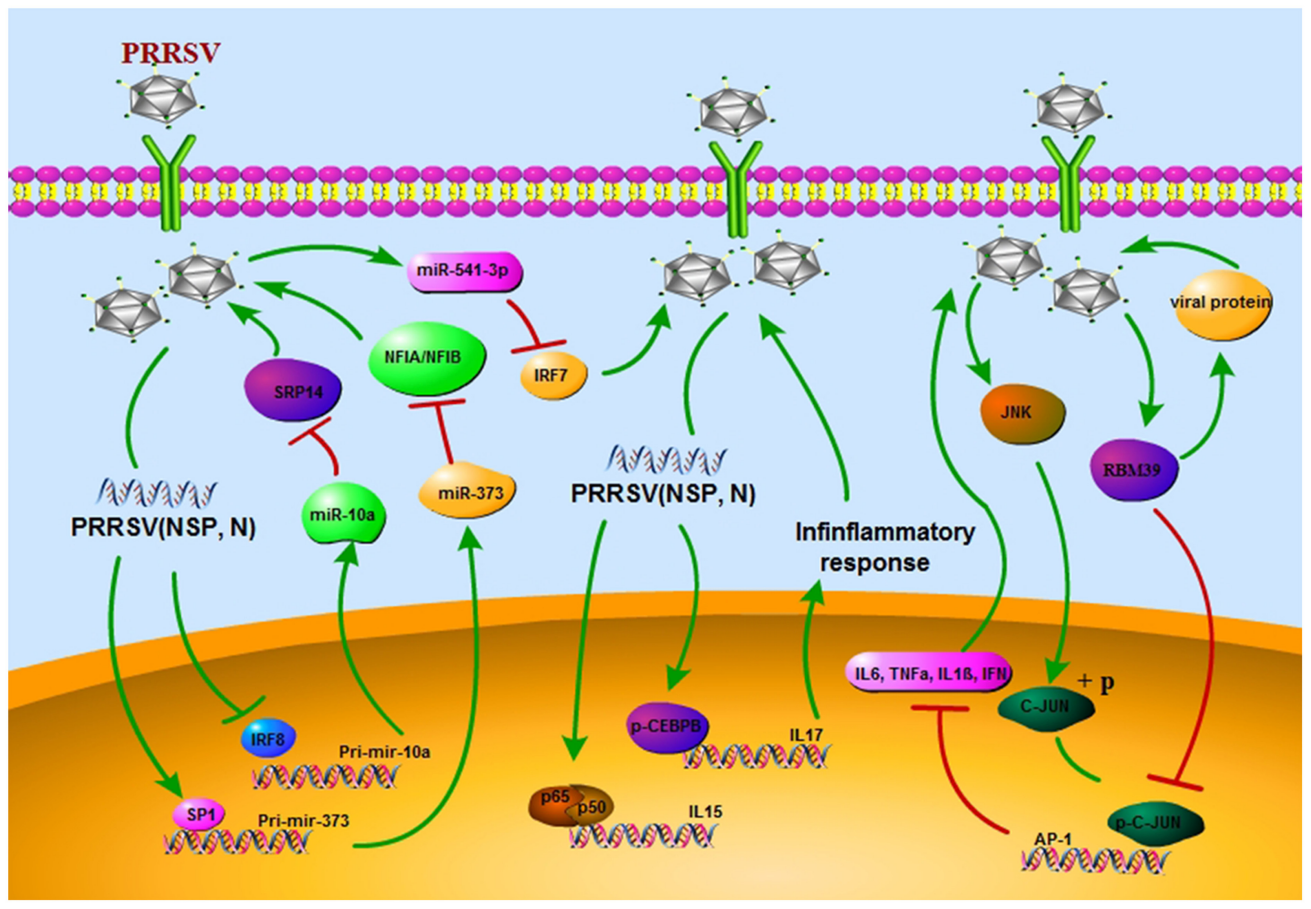
PRRSV escapes the host immune response through different strategies. First, the regulatory network of transcription factors/miRNAs promotes viral replication *in vivo*. Secondly, it acts on inflammatory factors, exacerbates inflammatory response. In addition, viral replication is promoted by nuclear localization and phosphorylation of transcription factors.

The PRRSV has greatly affected pig breeders around the world. Despite great advances in our understanding of PRRSV, no effective means to induce broad protective immunity is available. PRRSV infection affects both innate and adaptive immune responses by delaying the formation of neutralizing antibodies and deregulating cytokine expression. The study of TFs has improved our understanding of the underlying mechanisms for the regulation of abnormal gene expression. In the case of PRRSV infection, TFs mediate the structural and non-structural proteins of PRRSV for the regulation of inflammatory gene expression, immune cells, non-coding RNA and other related host factors and also for evasion of host immune response, which in turn promotes virus replication. TFs also help to clear viruses that are not eliminated by the host immune system and inhibit virus invasion (Figure 2). The use of small molecule drugs targeting specific TFs is widely used in the treatment of various human diseases; hence, due to their importance in many biological processes and their abnormal activity during PRRSV infection, TFs should be considered as future therapeutic targets.

## Author contributions

XY performed a literature search and then wrote the review article. XY, YY, and DX contributed intellectually to the general concept of the manuscript and flow of content. YL and PZ reviewed the manuscript and suggested and made relevant changes before submission. ZA checked the language and grammar. All authors have read and agreed to the published version of the manuscript.

## Funding

This work was supported by the Henan University of Science and Technology Research Startup Fund (13480095) and Henan Province Scientific and Technological Key Project (222102110445 and 222102110477).

## Conflict of interest

The authors declare that the research was conducted in the absence of any commercial or financial relationships that could be construed as a potential conflict of interest.

## Publisher's note

All claims expressed in this article are solely those of the authors and do not necessarily represent those of their affiliated organizations, or those of the publisher, the editors and the reviewers. Any product that may be evaluated in this article, or claim that may be made by its manufacturer, is not guaranteed or endorsed by the publisher.
